# Impact of Charged Particle Exposure on Homologous DNA Double-Strand Break Repair in Human Blood-Derived Cells

**DOI:** 10.3389/fonc.2015.00250

**Published:** 2015-11-11

**Authors:** Melanie Rall, Daniela Kraft, Meta Volcic, Aljona Cucu, Elena Nasonova, Gisela Taucher-Scholz, Halvard Bönig, Lisa Wiesmüller, Claudia Fournier

**Affiliations:** ^1^Department of Obstetrics and Gynaecology, Ulm University, Ulm, Germany; ^2^Department of Biophysics, GSI Helmholtz Center for Heavy Ion Research, Darmstadt, Germany; ^3^German Red Cross Blood Service Baden-Wuerttemberg – Hessen, Institute for Transfusion Medicine and Immunohematology, Johann Wolfgang Goethe-University Hospital, Frankfurt, Germany

**Keywords:** breakpoint cluster region, charged particles, chromosomal breaks, radiation damage response, DNA double-strand break repair, hematopoietic stem and progenitor cells, radiation-induced leukemia

## Abstract

Ionizing radiation generates DNA double-strand breaks (DSB) which, unless faithfully repaired, can generate chromosomal rearrangements in hematopoietic stem and/or progenitor cells (HSPC), potentially priming the cells towards a leukemic phenotype. Using an enhanced green fluorescent protein (EGFP)-based reporter system, we recently identified differences in the removal of enzyme-mediated DSB in human HSPC versus mature peripheral blood lymphocytes (PBL), particularly regarding homologous DSB repair (HR). Assessment of chromosomal breaks via premature chromosome condensation or γH2AX foci indicated similar efficiency and kinetics of radiation-induced DSB formation and rejoining in PBL and HSPC. Prolonged persistence of chromosomal breaks was observed for higher LET charged particles which are known to induce more complex DNA damage compared to X-rays. Consistent with HR deficiency in HSPC observed in our previous study, we noticed here pronounced focal accumulation of 53BP1 after X-ray and carbon ion exposure (intermediate LET) in HSPC versus PBL. For higher LET, 53BP1 foci kinetics was similarly delayed in PBL and HSPC suggesting similar failure to repair complex DNA damage. Data obtained with plasmid reporter systems revealed a dose- and LET-dependent HR increase after X-ray, carbon ion and higher LET exposure, particularly in HR-proficient immortalized and primary lymphocytes, confirming preferential use of conservative HR in PBL for intermediate LET damage repair. HR measured adjacent to the leukemia-associated *MLL* breakpoint cluster sequence in reporter lines revealed dose dependency of potentially leukemogenic rearrangements underscoring the risk of leukemia-induction by radiation treatment.

## Introduction

Radiation exposure increases the risk for acute myeloid leukemia (AML), as observed in atomic bomb survivors (1), occupational radiation workers (2, 3), and cancer survivors treated with radiotherapy (4). This is important especially in light of the increasing use of charged particles in cancer therapy (5, 6). Furthermore, a long-term leukemia risk for astronauts exposed to protons and high-energy charged particles during extended space travel is expected (7–9). As for all of these radiation scenarios densely ionizing radiation, such as charged particles or neutrons, contribute to the delivered dose, we need to understand whether densely ionizing radiation and photons differ in their impact on AML development.

Densely ionizing charged particles differ from sparsely ionizing photons in both physical characteristics and biological effectiveness (10). The greater effectiveness of densely ionizing charged particles is reflected in the severity of DNA lesions, which manifests both at the nanometer and the micrometer scale: DNA lesions are more complex and hence, more difficult to repair, as well as the complexity of chromosomal aberrations is higher (11, 12). In consequence, the number of unrepaired or misrepaired lesions and their transmission to the affected cell’s progeny, considered to be the basis for cancer induction, is greater for charged particles than for photons.

In the context of radiation exposure, induction of hematological malignancies, in particular of AML, was discussed to originate from error-prone repair of radiation-induced double-strand breaks (DSB) causing chromosomal rearrangements (13–16). Especially precarious targets for leukemic transformation are hematopoietic stem and/or progenitor cells (HSPC). HSPC are long-lived, self-renewed, and give rise to all types of mature blood cells and therefore are an ideal model system to study consequences of radiation exposure and the fate changes associated there with. On the other hand, mature peripheral blood lymphocytes (PBL) represent an extensively studied system in which cytogenetic damage has been established as a reliable biomarker of radiation late effects (17–19).

In our previous work, we studied the repair of DSB induced by photon radiation in the hematopoietic system (20, 21). We comparatively analyzed the capacity and quality of DSB repair in cycling human HSPC and PBL cultures mimicking exit from quiescence in response to stress conditions, such as infection or irradiation (22). Even though γH2AX signals and cytogenetic analysis suggested quantitatively similar DSB formation and removal after irradiation, we found substantial qualitative differences in DNA damage responses, i.e., differential use of DNA repair pathways. To dissect DSB repair mechanisms, we used our fluorescence-based assay system for extrachromosomal DSB repair (23), which has proven a valuable tool in various cell types including lymphoblastoid cell lines (LCL) derived from patients with genomic instability syndromes (24–26). Using this system, recombination of DSB can be detected after I-*Sce*I-endonuclease-mediated cleavage, but also independently of targeted cleavage by I-*Sce*I after various carcinogenic treatments including ionizing radiation (27–29). Application of this enhanced green fluorescent protein (EGFP)-based reporter system revealed a relative preference of error-prone non-homologous end joining (NHEJ), such as microhomology-mediated end joining (MMEJ) and single-strand annealing (SSA) in HSPC, as opposed to conservative NHEJ and high-fidelity homologous DSB repair (HR) in PBL. Furthermore, differential recruitment of repair proteins suggested a delay in the progress of the repair steps toward HR. We could identify differential NF-κB signaling as a critical molecular component underlying the observed differences: while in PBL, active NF-κB promotes HR and prevents compensatory accumulation of radiation-induced 53BP1 foci, in HSPCs, significantly reduced NF-κB activity and hence NF-κB target genes impedes accurate DSB repair.

To assess the effect of different radiation qualities in this study, we used the substrates HR-EGFP/3′EGFP or HR-EGFP/5′EGFP which detect both conservative and non-conservative HR or solely conservative HR, respectively, i.e., the very repair pathways which markedly differ in HSPC compared to PBL (20). Since radiation not only causes clean DSB but also generates base damage, single-strand breaks and complex DSB (12, 30), recombinative rearrangements, as monitored in our assay system, are ideal readouts to sense all these types of DNA lesions (29). The usage of differentially designed repair substrate plasmids allows discrimination between different repair mechanisms and repair qualities which is of major interest with regard to the repair of complex DNA lesions, such as are induced by charged particle radiation (11, 18, 31).

A refined repair assay variant integrates a highly fragile region within the mixed lineage leukemia breakpoint cluster region (*MLL*bcr), where cancer treatment-induced translocation sites predisposing to secondary leukemia have been found to cluster (29, 32, 33). Rearrangements involving the *MLL* gene are found in ~40% of therapy-related acute leukemias (33). Both chemotherapy and radiotherapy increase the risk factor for secondary malignancies of the hematopoietic system (34). Moreover, *MLL* rearrangements were identified after radiation exposure following the Chernobyl accident (35). Our own published data confirm preferential *MLL*bcr breakage compared to other sequences within the genome by γ-rays in both human HSPC and human PBL (20). In the current study, *MLL*bcr-based reporter cell lines were employed for the detection of radiation-induced chromosomal rearrangements. To this end, a 0.4 kb fragment of the *MLL*bcr sequence was introduced between the differentially mutated *EGFP* genes in the HR-EGFP/3′EGFP substrate. *MLL*bcr-based reporter cell clones were generated by stably integrating the substrate into the genome of the human myeloid leukemia cell line K562 and the human LCL WTK1 (29). The resulting K562(HR-EGFP/3′EFP-MLL) and WTK1(HR-EGFP/3′EFP-MLL) reporter cell lines represent more sensitive systems to study genotoxic treatment-induced (and thus likely also radiation-inducible) rearrangements.

The work presented here focuses on the impact of high LET compared to photon exposure on the induction and removal of DNA damage in immature and mature hematopoietic cells. Extra- and intrachromosomal reporter systems as described above were applied to compare maturity-dependent HR pathway usage and to analyze leukemia-associated rearrangements in reporter cell lines as a function of radiation quality.

## Materials and Methods

### Primary Cells

Hematopoietic stem and/or progenitor cells and PBL were isolated from peripheral blood samples of healthy donors, provided by one of us (HB). Donors provided written informed consent. The study was approved by the local advisory boards (approvals #329/10; #157/10; and #155/13). Donor treatment was performed with 10 μg/kg G-CSF per day for five consecutive days as described (36). HSPC were enriched by immuno-magnetically isolating CD34^+^ cells (MicroBead Kit, Miltenyi Biotech, Bergisch Gladbach, Germany) from G-CSF-mobilized donor blood as described (31). PBL were isolated from healthy donor buffy coats by Ficoll density-gradient centrifugation as described in Ref. (26).

Quiescent (G_0_-phase) HSPC and PBL were recruited into cell cycle prior to irradiation experiments by culturing in expansion media for 72 h at 37°C in a humidified atmosphere (95%). HSPC were kept in serum-free StemSpan SFEM medium supplemented with 100 ng/ml Flt-3 ligand (Flt3L), 100 ng/ml stem cell factor (SCF), 20 ng/ml Interleukin-3 (IL3), and 20 ng/ml Interleukin-6 (IL6) (Cytokine Cocktail CC100, both from StemCell Technologies Inc., Cologne, Germany). PBL were cultured in RPMI 1640 medium supplemented with 20% fetal calf serum (FCS), 3 mM l-glutamine, and 2% phytohemagglutinin (PHA) (components from Biochrom AG, Berlin, Germany).

### Cell Lines

In parallel to primary cells and as internal standards, we used the LCL 416MI and TK6, cultured in RPMI 1640 medium supplemented with 10% FBS, 1% penicillin/streptomycin, and 1% l-glutamine, as described before (25).

The human myeloid leukemia cell line K562(HR-EGFP/3′EFP-MLL) and the human B-LCL WTK1(HR-EGFP/3′EFP-MLL) were grown in suspension culture in RPMI 1640 medium supplemented with 10 and 12% FCS, respectively, and 100 U/ml penicillin and 100 μg/ml streptomycin (all reagents from Biochrom AG).

### Irradiation with Photons and Heavy Ions

Actively cycling cells were exposed to X-rays (16 mA, 250 kV, Seifert Isovolt DSI X-ray tube) or to γ rays (gamma irradiator, GSR D1, Gamma-Service Medical GmbH). Exposure of cells to heavy ions was performed at the heavy ion synchrotron (“Schwerionensynchroton,” SIS, GSI Helmholtzzentrum für Schwerionenforschung GmbH, Darmstadt, Germany).

At the time of photon exposure, cells were kept in medium in 5 ml tubes or 24-well plates with a dose rate of ~1 Gy/min. For heavy ion irradiation, the exposure with a monoenergetic beam or spread-out Bragg peak (SOBP) was performed, as described in Ref. (31). The parameters of the radiation exposure for the heavy ions used in this study are listed in Table 1.

**Table 1:** **Parameters for the heavy ions used**.

Ion	Energy (MeV/u)	LET (keV/**μ**m)	Track radius (**μ**m)^a^	Dose	Fluence^b^ (particles/cm^2^)	Hits per nucleus^c^
Nitrogen	130	40–65	243	2 Gy	2.4 × 10^7^	14 (HSPC)
						12 (PBL)
Carbon	114–158	60–85	262	2 Gy	1.72 × 10^7^	10 (HSPC)
						9 (PBL)
Titanium	1000	150	310	2 Gy	8.3 × 10^6^	5 (HSPC)
						4 (PBL)
Iron	1000	155	328	2 Gy	8.1 × 10^6^	5 (HSPC)
						4 (PBL)
Calcium	200	180	505	2 Gy	7 × 10^6^	4 (HSPC)
						3,5 (PBL)

*^a^The maximum range of delta electrons/track radius was calculated according to Ref. (37): *R*_max_ (μm) = 0.062 × E (MeV/u)^1.7^*.

^b^The fluence was calculated according to the formula: $D[Gy]=1.6\times 10^{-9}\times L_{\Delta }\left[\frac{keV}{\mu m}\right]\times \varphi \left[\frac{1}{cm^{2}}\right].$

*If SOBP irradiation was performed, the fluence of particles mostly contributing to dose deposition was calculated from the mean of the dose averaged LET*.

*^c^The hits per nucleus were calculated based on the geometric cross section, i.e., area of the cell nuclei (HSPC: 60 μm^2^; PBL: 50 μm^2^) and the fluence*.

### Premature Chromosome Condensation

At different time points after irradiation (0–9 h) radiation-induced breaks were measured in G_2_-phase cells by premature chromosome condensation (PCC) technique, as described elsewhere (38). Briefly, PCC was chemically induced by Calyculin A. Samples were processed as for metaphase analysis and stained with Giemsa, as described in Becker et al. (31). At least 50 G_2_-phase cells were analyzed per data point. In G_2_-phase cells, the total number of breaks was counted; chromatid and isochromatid breaks were scored as one and two breaks, respectively. In the following, we refer to the sum of both as “chromatid breaks.” A minor number of exchanges (≤5% of the breaks and comparable for both cell types), which appeared some hours after exposure, were scored as two breaks. The type of exchanges and the low fraction are comparable to previously reported ones (38).

### Quantitative Immunofluorescence Microscopy

At different time points after irradiation (1–24 h), cells were spun on cover slips, fixed with 3.7% PFA and permeabilized with 0.5% Triton followed by washing and blocking steps with PBS and 5% goat serum in PBS. Cells on cover slips were immunostained with primary antibodies anti-γH2AX (Ser139, clone JBW301, Millipore), anti-53BP1 rabbit NB100-304 (Novus Biologicals, Littleton, CO, USA) and with Alexa Fluo^®^555-conjugated secondary antibodies (Invitrogen). Nuclear counter staining was performed with DAPI and cover slips were mounted with VectaShield mounting media (Vector Labs, Burlingame, CA, USA). Immunofluorescence signals were visualized by an Olympus BX51 epifluorescence microscope equipped with an Olympus XC10 camera and acquired images automatically analyzed by CellF2.5_analysis software including the mFIP software (Olympus Soft Imaging System, Münster, Germany) or by Keyence BZ-II Analyzer software (Keyence, Neu-Isenburg, Germany).

### DSB Repair by HR in HSPC and PBL

Pathway-specific DSB repair analysis in HSPC and PBL was performed as described in Ref. (23, 26, 39). Briefly, actively cycling cells were transiently nucleofected with the DSB repair substrate HR-EGFP/5′EGFP (long homologies), detecting conservative HR, according to an Amaxa^®^ protocol (Human B Cell Nucleofector Kit; Human CD34^+^ Cell Nucleofector Kit; Lonza, Cologne, Germany) via electroporation (Bio-Rad Laboratories, Hercules, CA, USA). While DSB formation within the substrate is usually induced by co-nucleofection of the I-*Sce*I meganuclease expression plasmid pCMV-I-SceI, in the present study, the nucleofection mixture did not contain the expression plasmid. Instead, DSB were induced by exposing the cells 2–4 h after nucleofection to X-rays or heavy ions (carbon and calcium ions).

The assay monitors reconstitution of wild-type EGFP, so that EGFP-positive cells were quantified 24 h post-irradiation by the diagonal gating method in the FL1/FL2 dot plot (FACS Calibur^®^ FACScan, Becton Dickinson, Heidelberg, Germany), as described in Ref. (40). All nucleofections were performed in triplicates. The transfection controls additionally contained pBS filler plasmid (pBlueScriptII KS, Stratagene, Heidelberg, Germany) and wild-type EGFP expression plasmid for normalization of repair frequencies.

### Cell Lines (K562 and WTK1) with Stably Integrated *MLL*bcr Repair Substrate

Clones containing a single stably integrated copy of HR-EGFP/3′EGFP-MLL repair substrate were established from K562 and WTK1 cell lines, as described in detail in Ref. (29, 41). Briefly, cells were stably transfected with the *Xmn*I-linearized recombination vector pHR-EGFP/3′EGFP-MLLbcr.fwd. This DNA recombination substrate contains a 0.4-kb sequence of the genomic breakpoint cluster region (bcr) from the human *MLL* gene, which undergoes carcinogenic rearrangements in response to genotoxic treatment (42, 43). The cells were irradiated with X-rays or carbon ions. The reconstitution of wild-type EGFP (via conservative HR and SSA) was measured 24–48 h post-irradiation, as described in the previous section (see DSB Repair by HR in HSPC and PBL).

## Results

### Induction, Rejoining, and Manifestation of Radiation-Induced Chromatid Breaks

Induction and rejoining of radiation-induced breaks in PBL and HSPC were investigated with the PCC technique. Following *ex vivo* cultivation for 72 h, cells were irradiated with X-rays or charged particles (nitrogen, carbon, titanium, and calcium) in the LET range 45–180 keV/μm.

Regarding the induction level, it has to be taken into account that the number of chromatid breaks at 0 h (referred to as “initial breaks”) corresponds to the number of chromatid breaks detectable 5–15 min after exposure during which Calyculin A reaches the cells and prevents further repair. As shown in Figure S1 in Supplementary Material, the number of initial chromatid breaks increased in a linear dose-dependent fashion for both PBL and HSPC and also depended on radiation quality. For both cell types, the yield of chromatid breaks was similar. At the same physical dose (2 Gy), around 60–70 versus 40 chromatid breaks after irradiation with the different ions versus after X-ray exposure were measured in G_2_-phase cells, respectively.

**Figure 1:**
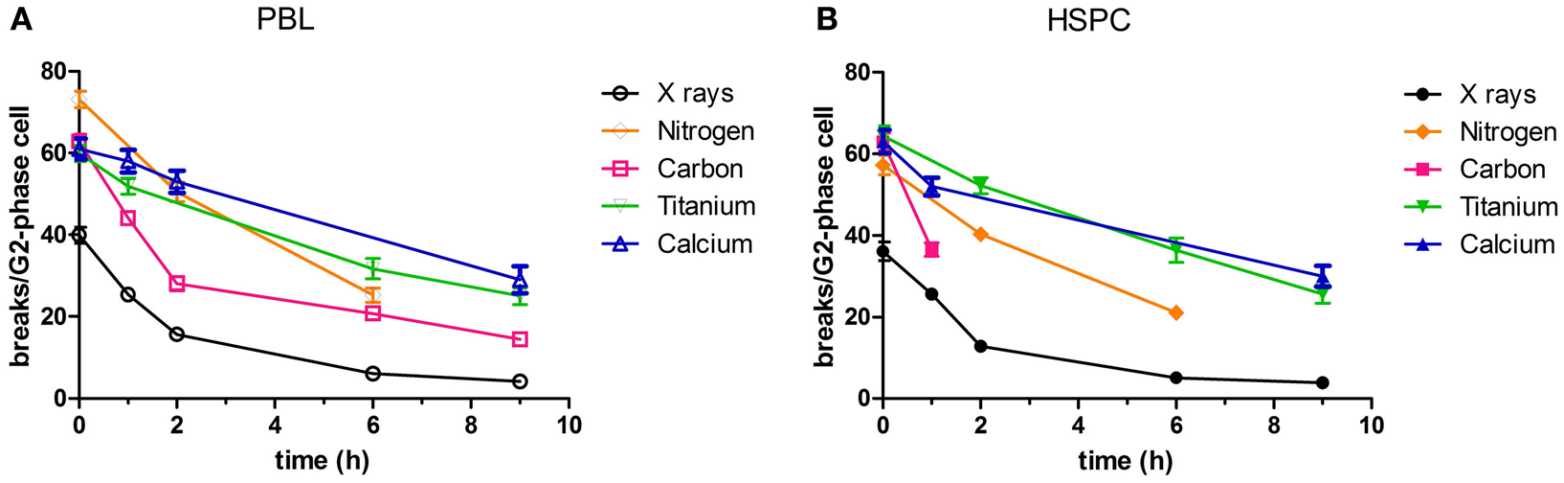
**Rejoining of radiation-induced chromatid breaks**. PBL and HSPC were stimulated for 72 h prior to irradiation with a dose of 2 Gy X-rays or charged particles. After irradiation, the cells were cultivated during the indicated periods of time. Charged particle exposure: nitrogen (45–65 keV/μm), carbon (60–85 keV/μm), titanium (150 keV/μm), or calcium (180 keV/μm). Premature chromosome condensation (PCC) was induced by Calyculin A. Slides were stained with Giemsa and at least 50 G_2_-phase cells were scored per data point. Numbers of independent experiments were for X-rays: *n* = 3; nitrogen, carbon, titanium, and calcium: *n* = 1. Mean values and SEM are indicated. For X-rays, SEM was calculated from mean values derived from independent experiments. For nitrogen, carbon, titanium, and calcium, SEM was calculated from values attributed to individual nuclei (>50). Connecting lines serve to guide the eye. Data for X-ray exposure are plotted from Kraft et al. (20). **(A)** PBL and **(B)** HSPC.

Rejoining of radiation-induced chromatid breaks was observed for 9 h after exposure (Figure 1). The number of chromatid breaks decreased with culture time with similar kinetics in both cell types. For X-ray irradiation, 1–2 h after irradiation more than half of the initial chromatid breaks had already been repaired. The time course of rejoining was similar for carbon ions (intermediate LET, 60–85 keV/μm, assessed in PBL) (Figure 1A), although the level of initial damage was higher compared to photons. However, following high LET exposure (calcium and titanium ions, 180 and 150 keV/µm, respectively), rejoining of chromatid breaks was slower. A major difference between the repair kinetics following exposure to X-rays and ions was that the number of chromatid breaks dropped to the level of controls, i.e., rejoining was finished almost completely within 9 h after irradiation (10% residual chromatid breaks, Figures 1A,B). In contrast, following irradiation with carbon ions a significant fraction of breaks remained unrejoined (23% residual chromatid breaks in PBL, Figure 1A), and after high LET calcium and titanium exposure, the level of residual damage was even higher (40–48% residual chromatid breaks, Figures 1A,B).

### Immunofluorescence Analysis of DSB Processing

**Figure 2:**
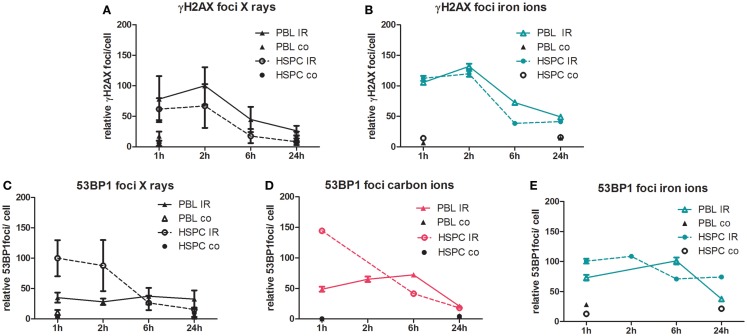
**Immunofluorescence analysis of DSB induction and repair after irradiation**. PBL and HSPC were stimulated for 72 h prior to irradiation without (co) or with (IR) a dose of 2 Gy of **(A,C)** X-rays, **(D)** carbon ions (60–85 keV/μm), or **(B,E)** iron ions (155 keV/μm). After irradiation, the cells were re-cultivated, fixed at the indicated time points, and immunolabeled for detection of **(A,B)** γH2AX or **(C–E)** 53BP1. Foci were scored by automated quantification from ~250 nuclei at each time point. Each number of foci per cell was normalized to the maximum mean value from the X-ray exposure time course data from the same experimental day. The 100% relative foci represent the following mean scores after X-ray exposure for γH2AX: 8 foci/cell (PBL/2 h) and 53BP1: 8 foci/cell (HSPC/1 h). Mean normalized values attributed to individual nuclei are shown with SEM (number of independent experiments for X-rays, PBL: *n* = 5; HSPC: *n* = 4; and heavy ions PBL and HSPC: *n* = 1).

To monitor DSB processing in response to treatment with ionizing radiation, we performed quantitative immunofluorescence microscopy of discrete nuclear foci, indicative of DNA lesions and in time course experiments of the accumulation and their removal (44). As shown in Figure 2, we measured γH2AX and 53BP1 foci in PBL and HSPC up to 24 h after radiation exposure with 2 Gy of X-rays, carbon (60–85 keV/μm), and iron ions (155 keV/μm). The different data sets were normalized to maximum foci values reached after X-ray irradiation to facilitate comparison with our recently published results (20). Using γH2AX as a DSB marker, formation and disappearance of foci was similar in both cell types for X-rays (Figure 2A), in agreement with our previous observations (20). Similar γH2AX curves for both cell types were also obtained following high LET iron ion exposure, but approximately threefold elevated levels of persisting DNA damage were detectable 24 h post-iron ion versus X-ray exposure (Figure 2B). Recently, we reported more pronounced accumulation of X-ray-induced nuclear 53BP1 foci in HSPC relative to PBL (20), which was confirmed here for X-ray and newly demonstrated for carbon ion exposure with intermediate LET (Figures 2C,D). However, with high LET iron ions, this striking difference between 53BP1 foci peak levels in HSPC and PBL disappeared (Figure 2E), mostly due to an increase of 53BP1 foci numbers in PBL 1 h post-irradiation with iron ions versus X-ray (Figures 2C,E). Concomitantly, the level of persisting 53BP1 foci 24 h post-irradiation was fivefold greater in HSPC following iron ion compared with X-ray exposure resulting in aggregate in very similar 53BP1 foci numbers 1–24 h post-irradiation. We obtained similar results as for iron ions with cells irradiated with high LET calcium ions (180 keV/μm, Figure S2 in Supplementary Material), i.e., 53BP1 foci curves for PBL and HSPC were comparable and the level of 53BP1 foci diminished only slightly over the time.

### Extrachromosomal DSB Repair Analysis Using Plasmid Reporter Systems

**Figure 3:**
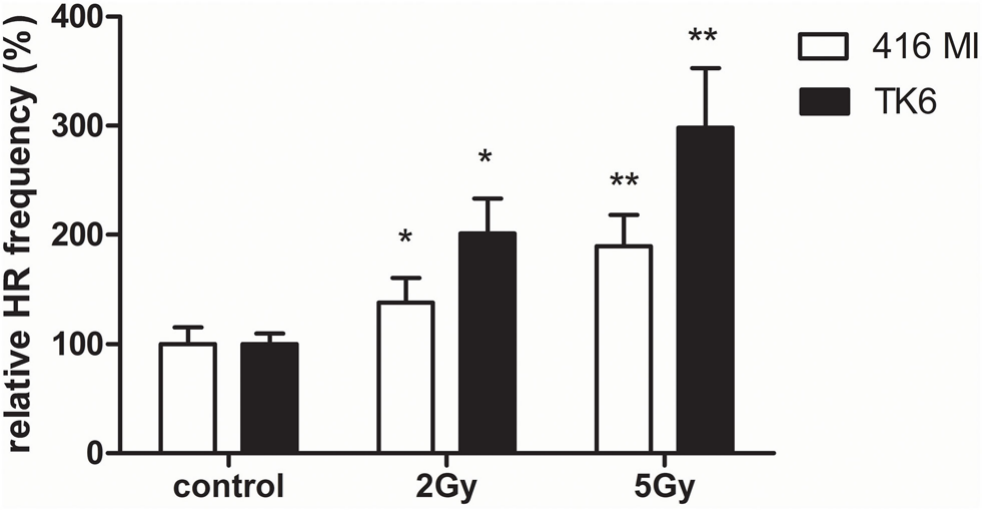
**Extrachromosomal DSB repair analysis in LCL following photon exposure**. The LCL 416MI and TK6 were transfected with HR-EGFP/5′EGFP, a DSB repair substrate which supports HR. Irradiation was performed with 2 or 5 Gy of photons (γ or X-rays). After subsequent incubation for 24–48 h, the fraction of EGFP-positive cells was quantified by flow cytometric measurement. Data were normalized to the non-irradiated control each. Mean values and SEM were calculated (416MI: *n* = 9–15 and TK6: *n* = 15–18). Statistically significant of differences between non-irradiated control and irradiated cells were calculated with the Wilcoxon matched-pairs signed rank test with *p < 0.05 and **p < 0.01.

In order to detect HR after exposure to X-rays and charged particles in PBL and HSPC, we used the EGFP-based plasmid reporter system described elsewhere (20, 23). In difference from our previous analyses engaging I-*Sce*I meganuclease for targeted cleavage, we tested if DSB formation within the substrate and subsequent repair can be induced by ionizing radiation. For this purpose, we transfected first the LCL 416MI and TK6 (25) either with the substrate HR-EGFP/3′EGFP (which supports both conservative and non-conservative HR) or HR-EGFP/5′EGFP (which detects conservative HR only), as these repair mechanisms were previously shown to be differentially active in PBL and HSPC (20). As demonstrated in Figure 3, in all LCL, exposure to photons (2 and 5 Gy) induced a significant dose-dependent HR increase. A dose-dependent effect was only detectable for the substrate HR-EGFP/5′EGFP, whereas for substrate HR-EGFP/3′EGFP, a general increase was observed (data not shown).

**Figure 4:**
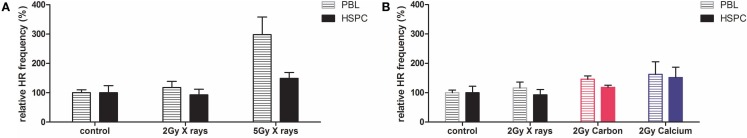
**Extrachromosomal DSB repair analysis in PBL and HSPC after irradiation with X-rays or charged particles**. PBL and HSPC were cultivated for 72 h, transfected with the DSB repair substrate HR-EGFP/5′EGFP which supports HR prior to irradiation with 2 or 5 Gy of **(A)** X-rays or **(B)** X-rays and charged particles (carbon ions, 60–85 keV/μm and calcium ions, 180 keV/μm). After incubation for 24 h, the fraction of EGFP-positive cells was quantified. HR data were individually normalized to the non-irradiated control representing 100% (PBL: 0.043 × 10^−2^ and HSPC: 0.048 × 10^−2^). Mean values and SEM are indicated (X-rays: *n* = 3 from one to three independent experiments, and carbon and calcium ions: *n* = 3 from one experiment). Mean values for non-irradiated controls and irradiated cells were compared with the Wilcoxon matched-pairs signed rank test; however, none of the differences reached statistical significance with *p < 0.05.

Based on these results, we investigated HR focusing on substrate HR-EGFP/5′EGFP in PBL and HSPC after photon or charged particle exposure by applying doses of 2 and 5 Gy (Figure 4). We observed a twofold higher 5 Gy radiation-induced HR frequency in PBL versus HSPC (0.2 × 10^−2^ versus 0.1 × 10^−2^), consistent with previous results for enzymatic cleavage (20). Interestingly, as can be seen in Figure 4A, X-ray irradiation led to relative increases in HR frequencies particularly in PBL even though in contrast to the LCL data (Figure 3), not reaching statistical significance with the limited number of experiments performed. Comparing radiation qualities at a single physical dose (2 Gy) revealed moderately, albeit statistically not significantly increased HR frequencies with higher LET (intermediate carbon ions and high LET calcium ions) (Figure 4B). Reminiscent of 53BP1 foci data, differences between HR frequencies were smaller in PBL and HSPC after calcium compared with carbon ion exposure.

In order to rule out that HR frequencies were influenced by potentially confounding factors in PBL and HSPC, the fraction of apoptotic cells and the cell cycle distribution were determined for X-ray and 60–85 keV/μm carbon ion exposures (Figure S3 in Supplementary Material). These radiation treatments increased the fraction of apoptotic cells (Figure S3A in Supplementary Material) and G_2_-phase cells (Figure S3B in Supplementary Material) in PBL and HSPC to a similar extent excluding a major role in cell type-specific HR activities.

### Radiation-Induced Intrachromosomal Recombination at the *MLL*bcr Sequence

The observed differences in extrachromosomal HR when comparing radiation qualities or cell types were mostly not statistically significant, which can be explained by the low probability of inducing a DSB in the target sequence of the reporter plasmid. The fraction of cells with one DSB was estimated at around 0.3%, taking into account the transfection efficiency, copy numbers, the size of the target sequence, and the estimated number of DSB per gray. As the fraction of cells with DSB is small and not all DSB are repaired by HR, we pursued an additional experimental strategy, using leukemia K562(HR-EGFP/3′EFP-MLL) and lymphoblastoid WTK1(HR-EGFP/3′EFP-MLL) cell lines (29) stably transfected with plasmid reporter comprising the highly fragile *MLL*bcr sequence (33). Exposure to different doses of X-rays or charged particles was performed. Highest doses (10 and 15 Gy X-rays, 5 Gy carbon and calcium ions) were excluded from the analyses because of associated cytotoxic effects as indicated by apoptosis-induction from sub G_1_ analysis (data not shown).

**Figure 5:**
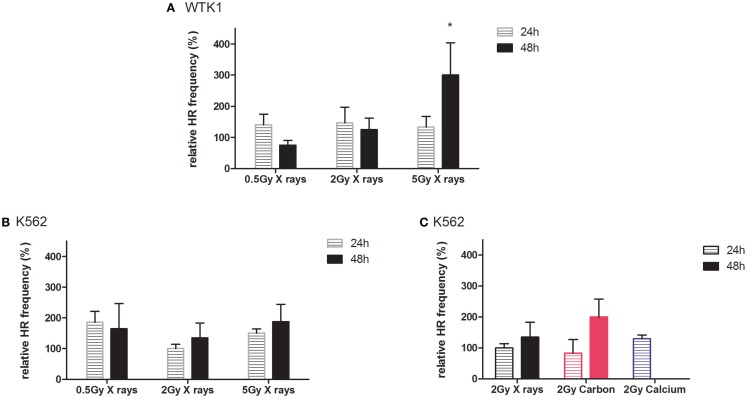
**Intrachromosomal DSB repair in WTK1(HR-EGFP/3**′**EFP-MLL) and K562(HR-EGFP/3**′**EFP-MLL) cells after irradiation**. Stably transfected WTK1(HR-EGFP/3′EFP-MLL) and K562(HR-EGFP/3′EFP-MLL) reporter cells were irradiated with **(A,B)** X-rays or **(C)** X-rays and charged particles (carbon ions, 60–85 keV/μm and calcium ions, 180 keV/μm). Radiation-induced breakage in the *MLL*bcr sequence within the chromosomally integrated HR reporter (HR-EGFP/3′EGFP-MLL) initiated HR events. After subsequent incubation for 24 or 48 h, EGFP-positive viable cells were analyzed within the total cell population by flow cytometric measurement. HR measurements were individually normalized to the unirradiated control representing 100%. Mean values and SEM are indicated (X-rays, WTK1 cells: *n* = 12 from four independent experiments; X-rays, K562 cells: *n* = 18 from six independent experiments; exposure to calcium and carbon ions: *n* = 3 from one independent experiment). Values for non-irradiated control and irradiated cells were compared with the Wilcoxon matched-pairs signed rank test with *p < 0.05.

Results from recombination measurements 24 and 48 h post-irradiation, indicating intrachromosomal rearrangements adjacent to the *MLL*bcr sequence, are shown in Figure 5. In general, radiation-induced stimulation of intrachromosomal HR was detectable in both cell lines (Figures 5A,B). Thus, we observed increased HR frequencies at least 48 h after X-ray exposure, except for one data point [0.5 Gy X-rays; WTK1(HR-EGFP/3′EFP-MLL)], displaying dose dependency and reaching statistical significance for 5 Gy in WTK1(HR-EGFP/3′EFP-MLL) cells. When comparing the same physical dose of 2 Gy in K562(HR-EGFP/3′EFP-MLL) cells applying X-ray versus ion exposure (Figure 5C), for carbon ions, more pronounced HR stimulation was observed after 48 h and for calcium, a trend toward enhancement was detectable after 24 h (48 h was not assessed). These data suggest that stably integrated *MLL*bcr sequences in a cell-based reporter assay can be useful for assessment of biological radiation effects.

## Discussion

Development of AML can be induced by ionizing radiation exposure (2, 3) and is contingent on the induction of specific chromosomal rearrangements and instability (45–47). For some time, we have known that chromosomal aberrations are mainly the result of DSB, which remain unrepaired or are not correctly repaired (48, 49). The frequency of misrepair depends on the type of damage, which can be simple or complex, and on the fidelity of the repair pathway chosen by the damaged cells.

The induction of complex DNA lesions is characteristic of ionizing irradiation; DNA and chromosomal damage induced by heavy ion irradiation is of higher complexity than photon induced damage due to the densely ionizing events occurring along the track of heavy ions. This leads to the occurrence of clustered lesions, i.e., closely spaced single-strand breaks or DSB that are frequently associated with additional types of lesions (50). These clustered lesions are difficult to repair and the level of unrepaired, persisting damage increases with ionizing density. Unrepaired lesions remain detectable as chromosome breakage (12), i.e., for terminal deletions, or lead to complex exchanges involving more than three chromosome breaks and multiple chromosomes (51, 52). Incorrect repair after high LET irradiation can cause point mutations (53) or enhance formation of intra- and interchromosomal exchanges (54–57). If the aberrations are lethal, these result in cell death or reduced clonogenic survival (58).

In our current study, we show a dose-dependent induction of chromatid breaks by X-ray irradiation (Figure S1 in Supplementary Material), and a more pronounced break induction and incomplete rejoining in response to high LET radiation qualities in PBL. We irradiated with five different ions (nitrogen, carbon, calcium, titanium, and iron ions) covering a LET range from 45 to 180 keV/μm (Figure 1A; Figure S1A in Supplementary Material). The level of residual damage at 9 h increased with LET, indicating a larger fraction of initial chromatid breaks refractory to rejoining after high LET compared to photon irradiation. This is in accordance with studies performed in different cell types (PBL, fibroblasts, epithelial cells) measuring residual chromosomal damage in mitotic or interphase cells (38, 52, 59–61). Of note, when comparing our results to reported data, one has to take into account that the absolute number of breaks depends on the protocol used for PCC technique (fusion with mitotic cells or Calyculin induced chromosome condensation) (62), the cell type (63), and the cell cycle stage of the irradiated and analyzed cells.

Up to now, rejoining of DSB in terms of chromosomal breaks by PCC has not been investigated for hematopoietic progenitor cells, i.e., HSPC. We demonstrate here similar, dose-dependent induction of chromatid breaks as for PBL and similarly decelerated rejoining after high LET exposure (Figure 1B; Figure S1B in Supplementary Material). Cytogenetic changes are considered a valid biomarker for cancer risk assessment (64), and have as such mostly been investigated in PBL isolated from blood of exposed individuals. The observed equivalent induction and repair of chromatid breaks in PBL and HSPC provides useful information because the cell of origin of leukemia is believed to be a transformed HSPC (65) and PBL are a commonly used model for assessment of chromosomal breakage and rejoining.

In good agreement with the cytogenetic data, using phosphorylation of H2AX as a DSB marker, we show that formation and removal of γH2AX foci is similar in both cell types for low and high LET radiation qualities (X-rays, iron ions). Based on the observed enhanced biological efficiency of titanium ions for the induction of chromatid breaks (Figure 1B), a higher induction of γH2AX foci by iron ions compared to photons might have been expected but was not observed (Figures 2A,B). We posit that this was most likely due to the limited resolution of γH2AX foci formed along a particle track (66, 67), although it is difficult to assess to what extent irradiation geometry would impact the irradiation of suspension cells.

As observed in the cytogenetic analyses, we also measured a higher level of persisting DNA damage after exposure to high LET iron ions compared to X-ray (Figures 2A,B). This characteristic of the high LET response, i.e., enhanced levels of γH2AX foci persisting after 24 h, was previously reported mainly in human fibroblasts, epithelial cells, and organotypic cultures (12, 68–72), while data for HSPC were not available.

Having identified an NF-κB-mediated decrease of HR in HSPC versus PBL in our preceding work (20), we assessed how this pathway is affected by radiation and damage quality in the different cell types. Using an EGFP-based reporter plasmid without expression of the cleaving enzyme, we found that extrachromosomal HR frequencies increased in immortalized lymphocytes (LCL 416MI and TK6) with X-ray dose (Figure 3). Consistent with previous results from enzyme-mediated cleavage, HR frequencies increased in X-ray-treated PBL and, less so, in HSPC (Figure 4). Interestingly, the difference between PBL and HSPC, best observed for 5 Gy X-ray, was not detectable for high LET calcium ions at 2 Gy despite a trend toward HR stimulation by 2 Gy heavy ion versus 2 Gy X-ray exposure.

Higher HR frequencies in PBL after irradiation were indeed expected from the previously obtained results for enzyme mediated cleavage. Comparing the same physical dose of 2 Gy X-ray and heavy ion irradiation, we further noticed a trend toward HR stimulation by heavy ion versus X-ray exposure. Interestingly, the difference between PBL and HSPC observed best for 5 Gy X-ray (Figure 4A) was no longer visible for high LET calcium ions (Figure 4B). This observation is likely not attributable to differences in cell cycle distribution between PBL and HSPC, because of a comparable radiation-induced cell cycle delay in G_2_ phase (Figure S3B in Supplementary Material).

In addition, we recently reported that the more pronounced formation of 53BP1 foci after X-ray-induced DSB in HSPC was a consequence of reduced NF-κB activity (20). Compromised NF-κB-mediated BRCA1-CtIP activation (73) can explain the observed relative shift to error-prone repair pathways in HSPC, possibly under participation of EXO1 nuclease as a resection factor (74, 75). This might also be relevant for particle radiation-induced DSB because we similarly found accumulation of 53BP1 foci after X-ray and carbon ion exposure (intermediate LET) of HSPC. However, this difference between immature and mature cells was lost after higher LET exposure (Figure 2E), consistent with similar HR frequencies after calcium ion irradiation (Figure 4B). Neutralization of the differences in 53BP1 foci numbers between PBL and HSPC was mostly due to elevated 53BP1 signals in PBL, suggesting incomplete HR repair of higher LET damage not only in HSPC but also in PBL. Results using LCL with stably integrated *MLL*bcr sequences further supported the impression of a dose and LET-dependent increase in HR frequencies (Figure 5). Even though further experiments are needed to generate a robust assay system to monitor the effects of different radiation qualities, our results provided clues for future directions (e.g., lentivirus-based integration of the reporter into primary cells of the hematopoietic system). Moreover, it underscored the detrimental potential of radiation-induced breaks to induce AML-related genome rearrangements at the *MLL*bcr in particular. Notably, HR was identified as a DNA repair pathway involved in *MLL*bcr rearrangements in response to replication stress, which can be induced in HSPC by stimuli, such as infection or irradiation (33).

Similarly, elevated 53BP1 damage levels and HR frequencies induced by high LET in PBL and HSPC match the concept that heavy ion-induced complex DSBs are predominantly repaired by HR and thus may exhaust the cellular HR machinery in both cell types (76). Conservative HR is limited to S/G_2_-phase cells (77–79) representing 40–60% of the primary cell populations in our study (Figure S3B in Supplementary Material). Other resection-dependent pathways, which are error-prone, have been suggested to contribute to the repair of complex damage (80). However, errors in repair can lead to chromosomal aberrations, in particular translocations (81, 82). Consistent with error-prone pathway usage in HSPC (20, 21), HSPC show a higher level of translocations compared to PBL at moderately enhanced LET (21, 31, 83). An additional explanation for similar HR frequencies in PBL and HSPC after high LET versus X-ray and carbon ion exposure could be earlier activation of NF-κB with increasing LET (84), which could compensate for the low intrinsic NF-κB activity in HSPC. In addition, activation of ATM, a prerequisite for NF-κB signaling, is also more pronounced with increasing LET (67).

Taken together, we could show that overall removal of radiation-induced DNA damage and chromosomal breaks is comparable for mature and immature cells of the hematopoietic system (PBL and HSPC). However, exposure to low and moderate LET reveals higher conservative HR in PBL versus HSPC, consistent with increased usage of low fidelity pathways during repair of enzyme-mediated DSB by HSPC. However, after exposure to high LET HR frequencies of PBL and HSPC are comparable, underlining the importance of HR for the repair of complex DNA damage for the outcome of the damaged cells (85, 86).

## Conflict of Interest Statement

Lisa Wiesmüller is an inventor of a patent on a test system for determining genotoxicities, which is owned by Lisa Wiesmüller. The remaining authors do not have any conflict of interest to declare.
