# Supplementary material for: Impact of Charged Particle Exposure on Homologous DNA Double-Strand Break Repair in Human Blood-Derived Cells
Source: Front Oncol. 2015 Nov 11;5:250. doi: 10.3389/fonc.2015.00250 (PMC4641431; doi:10.3389/fonc.2015.00250)
Supplement: Supplementary file 1 [file image_1.pdf]

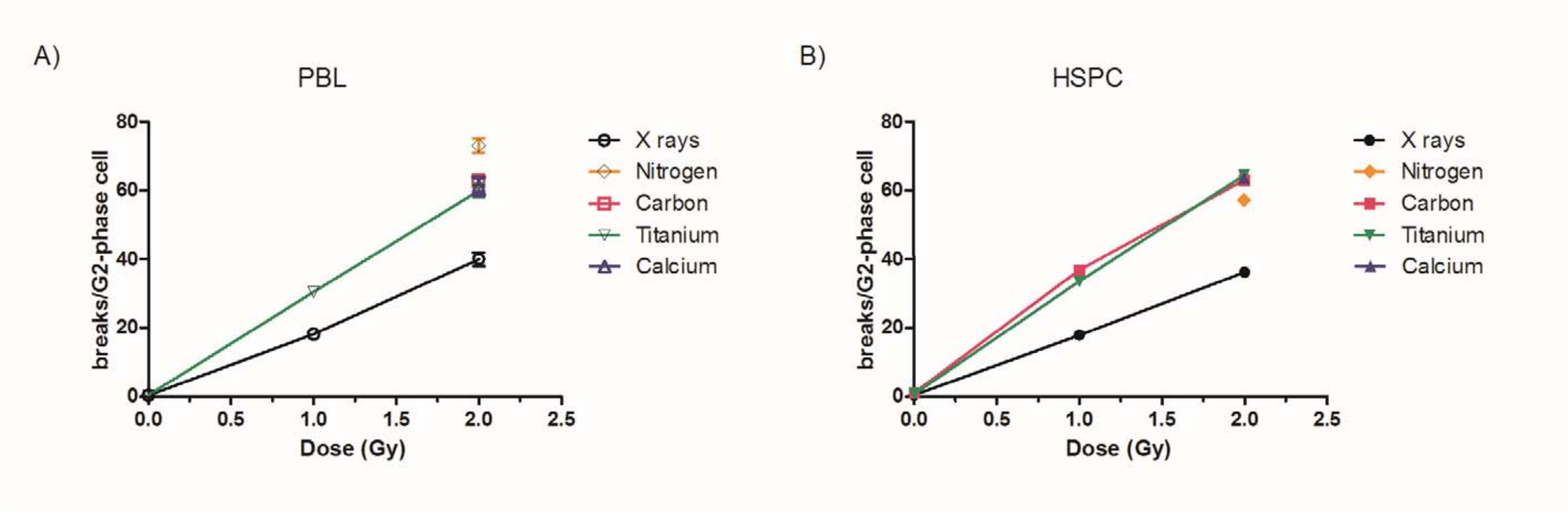

Supplementary figure S1: Initial induction of chromosomal breaks after radiation exposure.
PBL and HSPC were cultured for 72h followed by irradiation with X rays, nitrogen (45-65keV/µm), carbon (60-85 keV/µm), titanium (150keV/µm) or calcium (180keV/µm). Premature chromosome condensation (PCC) was induced immediately after radiation exposure by adding Calyculin A. Slides were stained with Giemsa and at least 50 G_2 phase cells were scored per data point. For X rays three independent experiments were performed, for nitrogen, carbon, titanium and calcium one experiment each. Mean values and SEM are indicated. If N=1 SEM was calculated from SD of all analyzed nuclei (>50). For X rays SEM was calculated from mean values derived from independent experiments. For nitrogen, carbon, titanium and calcium SEM was calculated from values attributed to individual nuclei (>50). Connecting lines serve to guide the eye.
(A) PBL
(B) HSPC
