# Supplementary material for: Impact of Charged Particle Exposure on Homologous DNA Double-Strand Break Repair in Human Blood-Derived Cells
Source: Front Oncol. 2015 Nov 11;5:250. doi: 10.3389/fonc.2015.00250 (PMC4641431; doi:10.3389/fonc.2015.00250)
Supplement: Supplementary file 2 [file image_2.pdf]

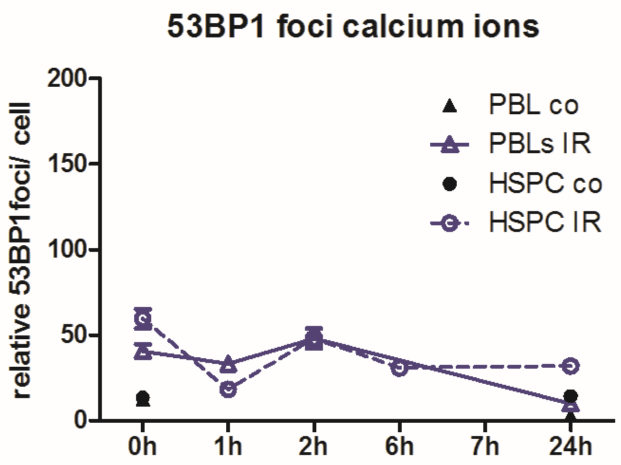

Supplementary figure S2: Immunofluorescence analysis of DSB induction and repair after calcium ion exposure. PBL and HSPC were cultured for 72h prior to irradiation without (co) or with (IR) a dose of 2Gy of calcium ions (180keV/µm). After irradiation, the cells were re-cultured, fixed at the indicated time points and immunolabeled for detection of 53BP1. Immunolabeled foci were scored by automated quantification in up to 250 nuclei for each time point. Single foci values per cell were normalized to the maximum mean value of the X ray time course data from the same experimental day. Mean normalized values from one experiment are shown with SEM.
