# Supplementary material for: Impact of Charged Particle Exposure on Homologous DNA Double-Strand Break Repair in Human Blood-Derived Cells
Source: Front Oncol. 2015 Nov 11;5:250. doi: 10.3389/fonc.2015.00250 (PMC4641431; doi:10.3389/fonc.2015.00250)
Supplement: Supplementary file 3 [file image_3.pdf]

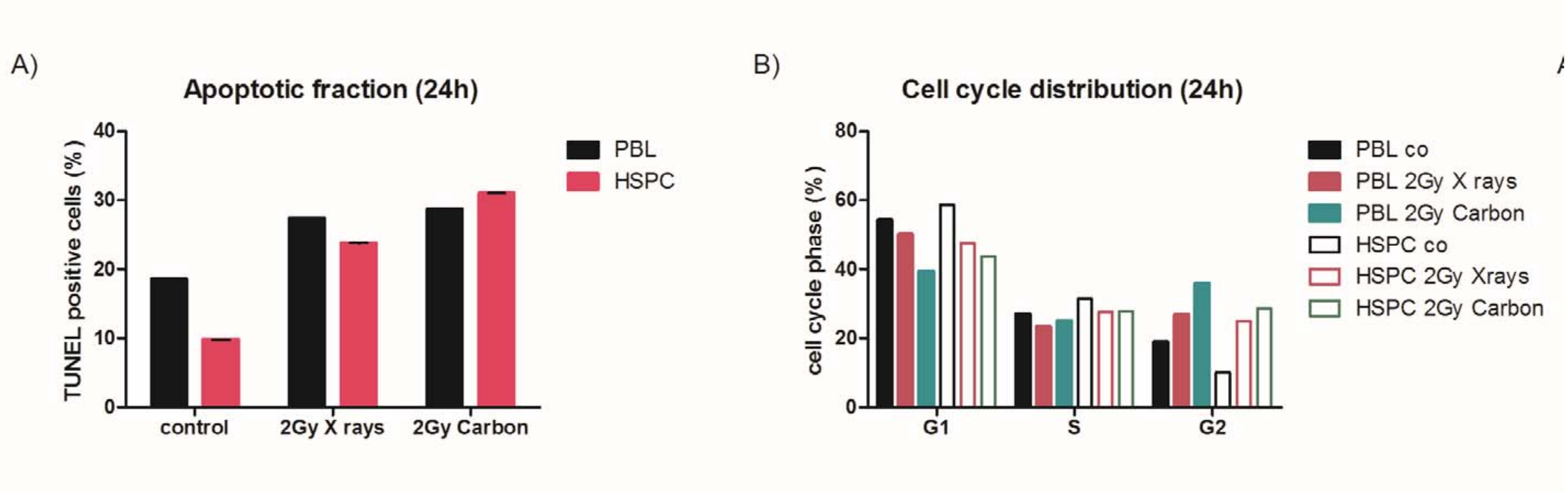

Supplementary figure S3: Analysis of apoptotic fraction and cell cycle distribution. PBL and HSPC were cultured for 72h followed by irradiation with a dose of 2Gy X rays or carbon ions (60-85 keV/µm) and reculturing for 24h. Cells were fixed and the percentage of apoptotic cells was determined by TUNEL staining (In Situ Cell Death Detection Kit Fluorescein, Roche, Germany) and flow cytometric analysis of at least 40.000 cells. DNA content was analyzed following DAPI staining (Becker et al. 2009). Percentages of living cells in G_1-, S-, and G_2-phase were determined by use of FlowJo software 7.6.5 (TreeStarInc, Ashland, USA) in one experiment. For the fraction of apoptotic cells, based on positive TUNEL staining, the (small) error bars were calculated assuming a binomial distribution (PBL: 0.0018-0.0019, for HSPC: 0.0015-0.0022 for HSPC).

(A) Apoptotic fraction of cells.

(B) Cell cycle distribution.
